# Sapropel as a Binding Material for Wood Processing Waste in the Development of Thermal Insulation Biocomposite

**DOI:** 10.3390/ma16062230

**Published:** 2023-03-10

**Authors:** Sigitas Vėjelis, Meruert Bolatkyzy Karimova, Tokzhan Kuangalyevna Kuatbayeva, Agnė Kairytė, Jurga Šeputytė-Jucikė

**Affiliations:** 1 Building Materials Institute, Faculty of Civil Engineering, Vilnius Gediminas Technical University, Linkmenų Str. 28, LT-08217 Vilnius, Lithuania; 2 T. Basenov Institute of Architecture and Civil Engineering, Satbayev University, 22 Satbayev St., Almaty 050043, Kazakhstan

**Keywords:** sapropel, biocomposite, wood processing waste, thermal conductivity, compressive stress

## Abstract

When developing new innovative building materials, their performance characteristics as well as their environmental friendliness are important. It is difficult to produce a fully ecological material for building envelopes, because there is a lack of ecological binding materials on the market, good binding materials are very expensive, and cheaper ones have poorer adhesive properties and performance characteristics. In this work, natural organic sapropel was used as an ecological binder. Before use, an organic sapropel was additionally mechanically activated. Its activation efficiency was evaluated on the basis of consistency and tensile strength. Sapropel activation increased its consistency from 112 to 168 mm and its tensile strength from 466 to 958 kPa. Wood processing waste was used as a filler for the thermal insulation biocomposite. Additionally, the wood waste was chopped to regulate the density and main performance properties of the biocomposite. The density of the biocomposite was also regulated using different amounts of sapropel and the degree of compaction of the composite mixture. In this work, the influence of the amount of sapropel, the level of compression of the biocomposite mixture, and the size of the wood waste particles on the thermal conductivity and compressive stress of the biocomposite was analyzed. It was found that the compression level had the greatest influence on both the compressive stress and thermal conductivity, up to 12 times and 43.3%, respectively.

## 1. Introduction

Most of the adhesives that are currently used contain toxic substances, pollute the environment, and cause serious human and animal health risks. Major groups of glue are produced on a formaldehyde and vinyl basis, covering 92% of the overall adhesive consumption [1].

In the construction materials industry, and especially in the production of engineered wood products, one of the most widely used binders are phenolic-based binders [2,3,4]. Formaldehyde-based binders are cheap and have a very strong adhesion to many materials, but they are very harmful—they have a carcinogenic effect [5,6]. Other synthetic binders are significantly more expensive or do not provide good adhesion properties. The production of fossil-based binders, especially cement, has a significant negative impact on the environment [7,8,9]. For these reasons, the need to replace synthetic and fossil materials with natural materials has increased in the construction materials industry in recent decades [10]. It should be noted that in recent decades, a great deal of attention has been paid to the development of new binders and adhesives. In reference [11], the authors created a very strong adhesive based on p-formylphenyl acrylate, achieving an average adhesion strength of up to 19.1 MPa. This adhesive was obtained with one-step synthesis, and it has the advantage of being simple to synthesize and use, can self-cure and adhere to a wide range of substrates, and maintains excellent adhesion strength in a variety of harsh environments. In reference [12], the authors used the basics of biomimicry and simulated the building process of a swallow nest. The developed composite had good thermal insulation properties and superior mechanical properties. To develop new material, the authors used bamboo scraps as the supporting framework and ethylcellulose molecular chains as the templates. In reference [13], it was shown that the wood binding interface may be generated by a crosslinking reaction between the active wood surface and the biomass-based adhesive (–CHO reacted with –NH2 to generate aminal and imine groups). An activated wood surface rich in –CHO groups was constructed by spraying a sodium periodate aqueous solution on a natural wood surface. In addition, microcrystalline cellulose was functionalized to obtain aminated cellulose, which was dissolved in an aqueous solution and used as a specific adhesive.

As a natural and environmentally friendly binder, sapropel can be used in the production of building materials [14,15,16]. Sapropel is an organic sediment of biogenic origin from freshwater bodies, formed over thousands of years after the death of planktonic or benthic organisms, which are basically processed by bacteria, insect larvae, worms, and mollusks in the absence of oxygen [17]. The rate of formation of the sapropel is only approximately 1–3 mm per year. The age of a sapropel layer several meters thick can span 10,000 years or more. One of the most characteristic features of sapropel is its colloidal structure. Organic colloids can absorb a lot of water, from 70% to 97%. Due to its colloidal structure, the sapropel has low filtration [18,19]. Sapropel also has specific properties: it dries slowly, evaporating water with difficulty, and when dry, it becomes completely hard, even when ground into powder, and does not absorb water (except for the carbonated variety) [17,20]. After freezing, the sapropel becomes fluffy and dries relatively quickly to 30–35% humidity, but loses its binding properties [20,21,22]. 

Sapropel can be used as a binder for various wood waste, unused waste from the paper and cardboard industry, flax processing by-products, degraded peat, and similar raw materials. Sapropel is a good substitute for protein-based glues, for example, albumin, and the possibilities to replace proteins [19] would be a significant benefit of the application of sapropel.

Sapropel research has received a lot of attention over the past several decades. Bogush et al. [23] indicate that sapropel studies are divided into two areas, fundamental and practical. The fundamental field includes paleoclimatic reconstructions, geochemical and biochemical studies, and the practical field, namely use in agriculture, balneotherapy, construction, and industry. According to the composition, the sapropel can be divided into three types, organic, organic-mineral, and mineral [24]. The composition of sapropel depends not only on the possibilities of its use but also its properties. Sapropel with an organic content of more than 85% and nitrogen of more than 3.3% can be used for the production of the sapropel binder [19]. The organic part of the sapropels contains from 3% to 11% bitumen, up to 40% humic substances, and other biologically active substances [20]. The bitumen components of the sapropel attract particular attention because they have high bactericidal, bacteriostatic, and antioxidant activity [25]. The authors indicate that the biobased composite material, where sapropel was used as a binder, shows one of the highest microbiological stability results. Furthermore, fungi and other organisms were not detected in the samples.

Although the resources of sapropel in the world are large, their use for the production of building materials is still stagnant. Several scientific studies have been conducted to demonstrate the suitability of sapropel in the production of building materials. As researchers [15] pointed out, the sapropel itself has a high bind ability, as well as shape-holding capacity, adhesive properties, and plasticity. Building materials made with sapropel can be cured in three ways: drying under natural conditions, with thermal treatment, or under high pressure to remove most of the water. Obuka [19] indicates that using sapropel as a binder without thermal processing is beneficial because it has low embodied energy and low CO_2_ emissions, making it very appropriate for use as an ecological insulation material with low environmental impact. With sapropel, the author produced two types of biocomposites: a sapropel-wood fiber composite and a sapropel-birch wood sanding composite. The density of these biocomposites was 319 and 470 kg/m^3^, the thermal conductivity 0.060 and 0.061 W/(mK), and the compressive stress 0.19 and 0.67 MPa, respectively. In [16] the sapropel-hemp shives composite has been obtained by mixing three main components in cyclic mixers: hemp shives, sapropel, and water in certain proportions. The prepared mixture was compacted in molds and initially dried in the laboratory under (19 ± 2) °C ambient conditions for 3–4 days, depending on the amount of binder. After this period, the specimens were removed from the moldings and continued to dry in the laboratory for 4–5 weeks until a constant weight of the specimens was achieved. Thermal conductivity at a measurement temperature of 10 °C varied from 0.046 to 0.054 W/(mK) at the density of the materials 180–200 kg/m^3^.

Another author [26] prepared biocomposites for building from hemp shive, sapropel, and paper production waste using thermal treatment. The specimens were cured in two stages: at 190 °C for 3 h and at 160 °C for 18 h. The author indicated that the cured specimens at 190 °C had the lowest water absorption. The compressive stress of the biocomposites made with the sapropel binder varied from 0.90 to 2.02 MPa and the density from 267 to 361 kg/m^3^. Meanwhile, the thermal conductivity varied from 0.059 to 0.068 W/(mK). Before the test, the specimens were conditioned for 72 h at (50 ± 5)% relative air humidity and (23 ± 5) °C temperature.

A new contribution of the work is the evaluation of the numerical values of the sapropel consistency and binding parameters, the evaluation of the influence of wood waste particles on the density and thermal conductivity, and the evaluation of the influence of the sapropel content, size of the wood waste particles, and compression of the mixture on the thermal conductivity and compressive stress of the created biocomposite. In this work, using sapropel as the binder and wood processing waste as the filler, a new biocomposite was created, primarily for thermal insulation but also with high strength properties.

## 2. Materials and Methods

### 2.1. Materials

Sapropel was taken from the bottom of the lake in the autumn, when the water temperature was 14 °C. Sapropel was taken from a depth of 3–5 m using special equipment, a modified bathometer mounted on a telescope tube. The bathometer is a hydrological instrument, a sampler for taking water samples from various depths of the reservoir. A bathometer is a specially adapted vessel, with valves to close under water at a given depth. In this way, the sample is taken accurately from the specified depth without mixing it with materials from other depths. A total of 200 L of sapropel were taken for the tests. The collected sapropel was stored in closed containers at a temperature of (20 ± 5) °C. The characteristics of the sapropel used in the study are presented in Table 1. The water content of the sapropel was determined by drying it in a drying oven at 105 °C according to EN 12570 [27], and the organic matter content was determined by heating the dried sapropel in an oven at 500 °C according to EN 13820 [28].

In order for the sapropel to have a good consistency and good binding, it was additionally mechanically activated. Mechanical activation is required to activate the bonds of the colloid system [26]. For this purpose, an industrial mixer was used with a vessel. The mixer speed was 12,500 rpm. The duration of the sapropel activation was varied from 1 to 3 min to assess the activation efficiency. The consistency of the sapropel was determined by the slump test. Tests were carried out on nonactivated sapropel and activated sapropels for different durations. The activated sapropel was placed in porcelain containers for different periods of time and cured for 24 h at 170 °C in a drying oven. The hardened sapropel was removed from the containers and regular-shaped specimens of approximately (30 × 30 × 15) mm in size were cut. The specimens were glued to metal plates with polyurethane glue and cured for 24 h in a conditioning room at a relative humidity of air (50 ± 5)% and a temperature of (23 ± 5) °C. The prepared specimens were fixed between the grips of the press and the maximum breaking load was recorded. The strain rate for the tensile was 10 mm/min. The tensile strength of the hardened sapropel was calculated automatically based on the measured area of the specimen and the maximum load, according to Formula (1) [29]:

(1) $$\sigma_{mt}=\frac{F_{m}}{A}$$

where *F_m_* is the maximum recorded tensile force, in kN; *A* is the cross-sectional area of the test specimen, in m^2^.

Three specimens with different durations of the activation of the sapropel were used for the tests.

The wood processing waste was taken from a wood processing workshop. Pine wood processing waste was used for the tests. In the production workshop, all generated wood waste was additionally crushed with industrial shredders and packed in polyethylene bags. To avoid uneven wood particles in the laboratory, wood processing waste was additionally crushed with a hammer mill with sieves of different mesh sizes. Wood waste particles of three sizes were used for the tests. The granulometric composition of the wood waste particles is presented in Table 2.

**Figure 1:**
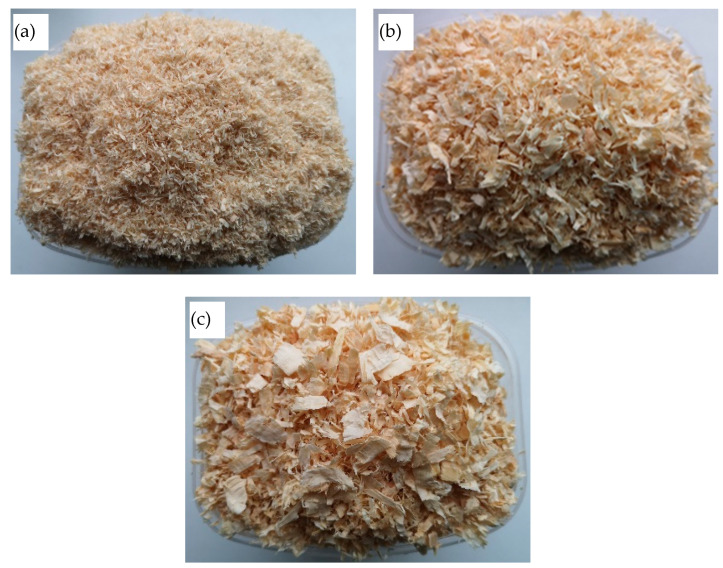
Image of the wood waste particles after being crushed using different sieve sizes, mm: (**a**) 5; (**b**) 10; (**c**) 20.

The image of the wood waste particles after being crushed in a mill using different sieves is presented in Figure 1.

The bulk density of the wood waste of different fractions was determined in a cylindrical metal container. Wood waste was poured into a 5 L container of known mass from a height of 15 cm until the container was filled to overflowing. With the help of a metal ruler, the pile was scraped off and the container with the wood waste was weighed. The test result was determined from three measurements. Based on the known weight and volume of wood waste, the bulk density of the material was calculated according to Formula (2):

(2) $$\rho_{b}=\frac{m}{V}$$

where *m* is mass of wood waste particles, in kg; *V* is the volume of wood waste, in m^3^. 

### 2.2. Preparation of Specimens 

The mixtures for the formation of biocomposites were prepared from mechanically activated sapropel and wood waste in various ratios. The components of the mixture were mixed in a forced mixing mixer. The prepared mixtures were placed in a pre-prepared metal mold, pressed to the intended limit, and cured in a drying oven for 6 h at 170 °C. A total of nine different mixtures were prepared—three mixtures of each wood waste fraction with three different amounts of sapropel. Each prepared mixture was compressed at four different compression levels during the preparation of the biocomposite. The compositions and compression levels of the mixtures used for the biocomposites are presented in Table 3.

### 2.3. Tests Metods

Thermal conductivity studies were performed on bulk wood waste and the prepared biocomposites. Bulk wood waste was poured into a pre-prepared mold, the edges of which were made of a rigid polystyrene foam and the bottom of which was made of perforated polyethylene. The internal dimensions of the mold were (250 × 250) mm and the height was 50 mm. The form was filled with wood waste in a pile, so that the surface of the formed specimen could be additionally pressed in order to avoid bad contact with the measuring plate. The compression load was 100 Pa. The density of the wood waste obtained in the mold was very close to the bulk density. The (300 × 300 × 50) mm specimens were prepared for thermal conductivity studies of biocomposites. Before testing, all specimens were conditioned for at least 72 h in an environment of (50 ± 5)% RH and (2 ± 23) °C temperature. Thermal conductivity was determined using a Fox 304 LaserComp (TA Instruments, Newcastle, DE, USA) heat flow meter instrument. The mean temperature of the measurements was 10 °C, and the upper and lower temperatures of the device measurement plates were 0 and 20 °C, respectively. Measurements were made in accordance with the requirements of EN 12667 [30].

The compressive stress was determined at 10% deformation, because the specimens do not disintegrate during compression but are compacted. Compressive stress was determined according to the requirements of EN 826 [31] and automatically calculated according to Formula (3):

(3) $$\sigma_{10}=10^{3}\frac{F_{10}}{A_{0}}$$

where *F*_10_ is the force corresponding to a strain of 10%, in N; *A*_0_ is the initial cross-sectional area of the specimen, in mm^2^, and *σ*_10_ is the compressive stress at 10% strain, in kPa.

Specimens of size (100 × 100 × 50) mm were prepared for testing. The rate of loading was 5 mm/min. Before the test, the specimens were conditioned for at least 72 h in an environment with (50 ± 5)% RH and a temperature of (2 ± 23) °C. The tests were performed using a Hounsfield H10KS (Hounsfield. Ltd., Salfords, UK) universal press. Three specimens were used for the tests of one composition. 

The effectiveness of the contact zones between the wood waste particles and the sapropel was evaluated using a JEOL JSM-7600F scanning electron microscope (JEOL, Tokyo, Japan). To analyze the structure of the composites, specimens of 30 × 30 × 30 mm were prepared.

## 3. Results and Discussion

### 3.1. Sapropel 

The results of the tests on the consistency of the mechanically activated sapropel are presented in Table 4.

**Figure 2:**
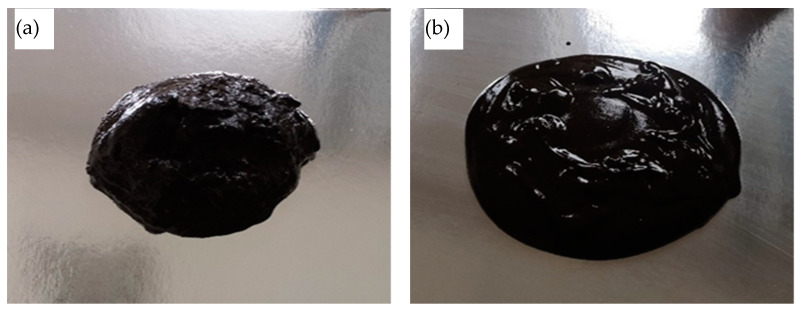

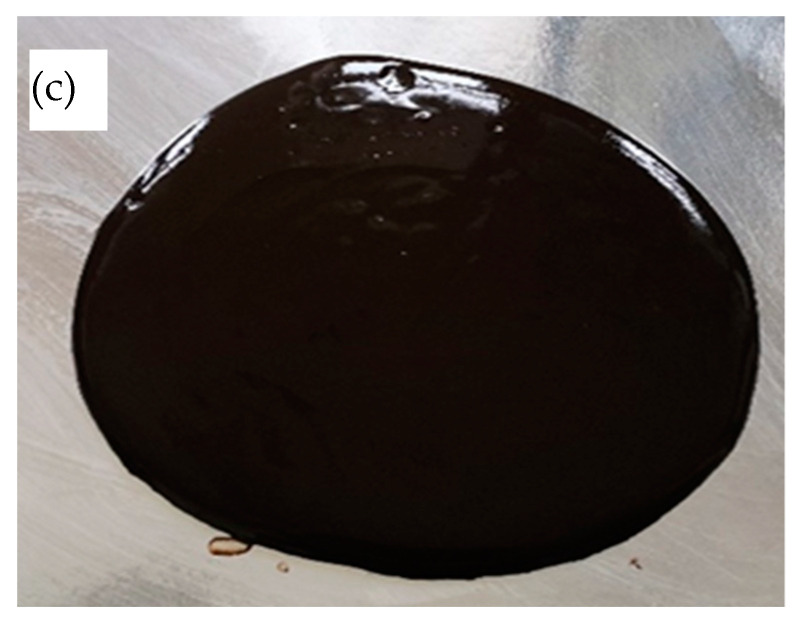
Spread shapes of a sapropel: (**a**) unactivated; (**b**) mechanically activated 1 min; (**c**) mechanically activated 2 min.

The results of the study show that mechanical activation of the sapropel increases its consistency by more than 60%. Furthermore, the consistency of the activated sapropel depends on the duration of activation. After 1 min of activation, not only is the consistency of the sapropel lower than when activated for 2 or more minutes, but visually (see Figure 2) it does not look like a homogeneous mass. The appearance of an inactive sapropel visually looks like a thick mass. During mechanical activation, the metastable state of the colloidal system is not only destroyed, but also the incompletely decomposed small particles in the sapropel mass, such as the wood remains, plant roots, etc., are crushed.

**Figure 3:**
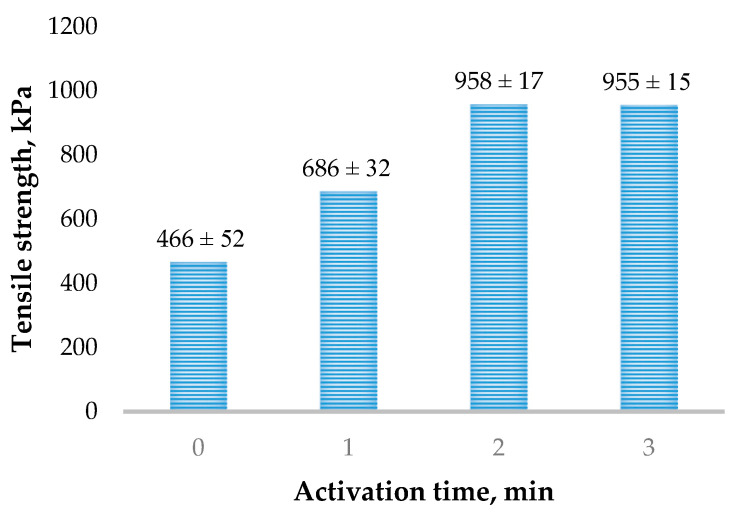
Tensile strength of the sapropel activated for different durations.

The assessment of the adhesive bonds of the sapropel is represented graphically in Figure 3. Analysis of the results shows that the sapropel mechanically activated for 2 and 3 min has the best binding properties. The tensile strength of the mechanically activated and hardened sapropel is approximately two times higher than that of the mechanically unactivated sapropel and approximately 1.4 times higher than that of the mechanically activated for only 1 min. The variance of the results of the tensile strength of the cured specimens prepared from non-activated sapropel and activated for 2 min was evaluated by the *t*-test. The obtained *t*-value (12.72) exceeded the critical value (2.78) and this confirmed that the results were significantly different. This indicates that mechanical activation significantly increases the adhesive properties of the sapropel.

**Figure 4:**
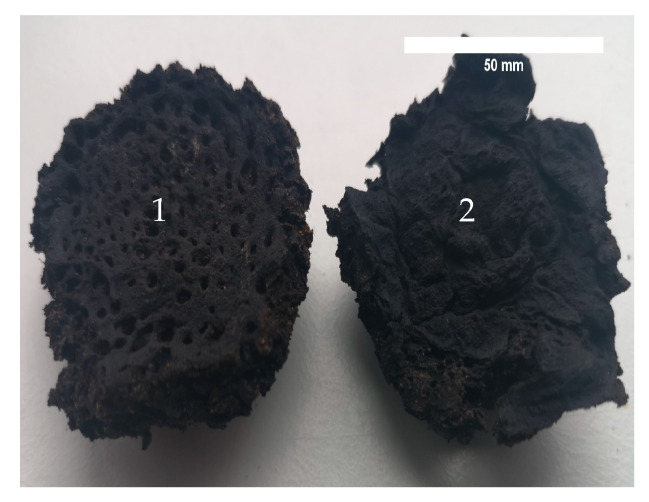
View of the hardened sapropel: 1—not activated; 2—activated for 3 min.

Figure 4 shows a view of the hardened sapropel. It can be seen visually that the unactivated sapropel hardens by forming a large-pore structure, while the mechanically activated sapropel hardens by forming a dense closed-pore structure. It is likely that similar hardening principles work during the formation of biocomposites, so, in the case of using unactivated sapropel, some of the fillers remain uncovered by the sapropel and homogeneous joints do not form.

The previously examined works of scientists [17,19,20] do not provide more detailed information on the methods and durations of sapropel activation, so it is not clear whether the sapropel was activated. Balčiūnas [26] used mechanical activation in his research, but the duration and effect of activation were not analyzed.

### 3.2. Wood Waste 

**Figure 5:**
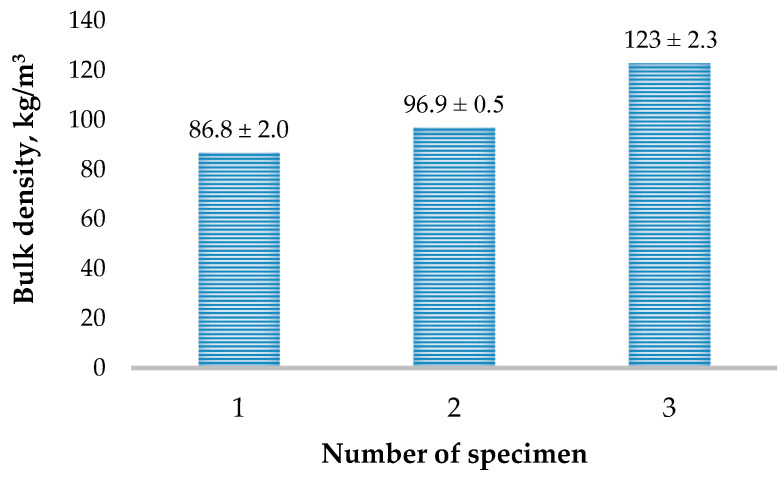
Results of the bulk density of wood waste, when fraction, mm: 1—0/20; 2—0/10 and 3—0/5.

In Figure 5, the results of the studies on the bulk density of the wood waste particles of different sizes are presented. The results of studies on the bulk density of wood waste of different sizes show a difference of more than 40%. The largest fraction has the lowest density, and the smallest fraction has the highest density. The higher density is determined by the greater number of fine particles in the fine fraction (see Table 2) and the formation of finer air gaps between the particles themselves (Figure 1).

**Figure 6:**
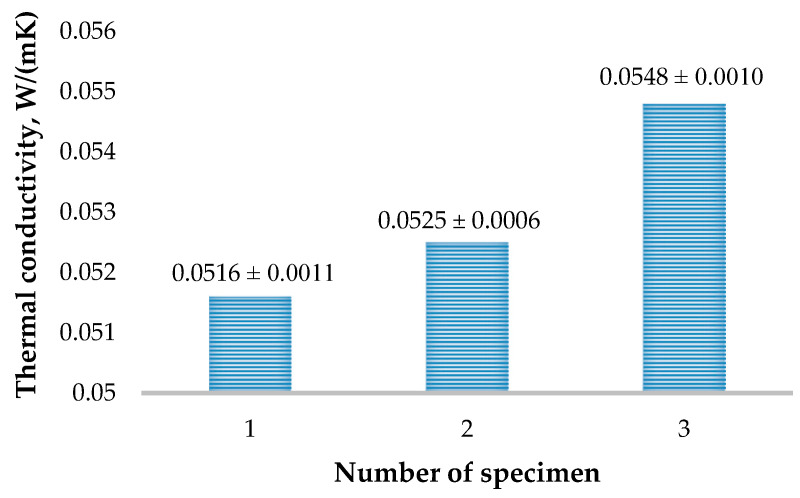
Thermal conductivity studies of wood waste, when fraction, mm: 1—0/20; 2—0/10 and 3—0/5.

In Figure 6, the results of the thermal conductivity of the wood waste particles of different sizes are presented. The research results show the difference in thermal conductivity between the different particle sizes and different specimen densities. The wood waste with the lowest density and the highest fraction (0/20 mm) has the lowest thermal conductivity, and the highest density particles (0/5 mm) have the highest thermal conductivity. Generally, thermal conductivity for low-density materials is determined by the size of the air gaps between particles, and for materials with a higher density by the amount of contact zones between the solid particles. In our case, the difference in thermal conductivity is small and, based on a detailed analysis of heat transfer methods [32], it can be said that the number of contact zones, rather than the size of the air gaps, has the greater influence.

The thermal conductivities of wood waste presented in the works of other authors are similar to the research conducted in our work [33]. The thermal conductivity of the wood waste determined by the authors ranged from 0.046 to 0.054 W/(mK). The authors particularly emphasized the negative effect of particles smaller than 0.315 mm on thermal conductivity. In our work, there was no large amount of fine particles due to additional crushing, because the fine particles were retained in the air filter of the mill.

### 3.3. Thermal Conductivity and Compressive Stress of Biocomposite

**Figure 7:**
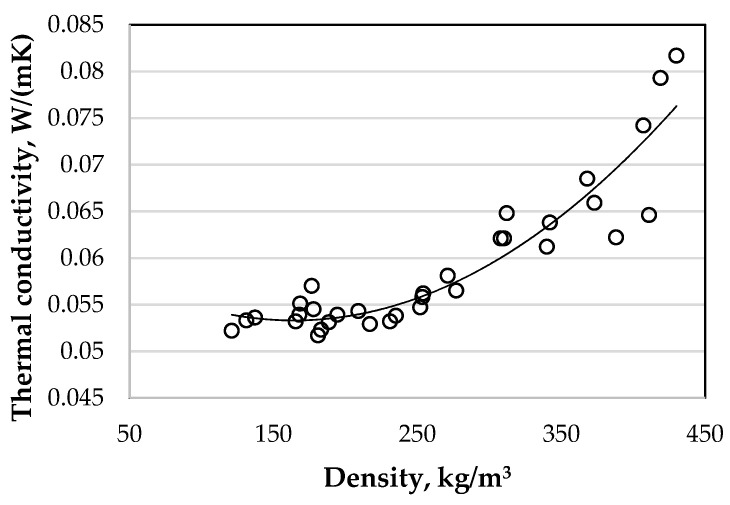
Dependence of the thermal conductivity of the biocomposite on its density.

Using wood waste particles of different sizes, different levels of compression, and different amounts of binder (Table 2), a whole series of specimens was prepared to evaluate the thermal conductivity and compressive strength. The prepared specimens were characterized by a large difference in density. The density of the specimens for the thermal conductivity tests varied from 87 to 430 kg/m^3^ and the thermal conductivity from 0.0496 to 0.0817 W/(mK). The results of the thermal conductivity tests are presented in Figure 7.

In Figure 7, a large spread of results cannot be seen, so it is difficult to judge the effect of various factors on the thermal conductivity of the biocomposite. To more accurately evaluate the influence of the size of the filler fraction, the compression level of the molding mixture, and the amount of sapropel in the mixture on the thermal conductivity of the biocomposite, we grouped the results. The analysis of the grouped results showed that the fine filler has a significant effect on the thermal conductivity of the biocomposite. When using the fraction fillers of 0/5 mm, the thermal conductivity coefficient of the biocomposite of the same compression and composition is 2–26% higher than when using the fraction fillers of 0/10 or 0/20 mm. The smallest difference is observed at the lowest biocomposite density and 20% compression, and the highest difference is observed at the highest biocomposite density and 80% compression. When comparing biocomposites formed from fraction fillers of 0/10 and 0/20 mm, there is no difference in the thermal conductivity, or if there is, the difference is insignificant. The analysis of the results shows that in all cases, using 0/5 mm fraction fillers, the density of the biocomposite is 1.31–1.55 times higher, which explains the higher thermal conductivity values of the formed biocomposites. When comparing the density of biocomposites, when fillers of fractions 0/10 and 0/20 mm are used, the density differences in all cases are only 1.06–1.09 times.

Increasing the amount of sapropel in the biocomposite mixture is of relatively minor importance. By increasing the amount of sapropel in the mixture three times, the thermal conductivity of the cured biocomposite increases only from 1.5% to 10.1%. Additionally, increasing the amount of sapropel is more important for the biocomposites made from the fraction 0/5 mm. For the biocomposites made from the 0/10 fraction, the maximum increase in thermal conductivity is observed by 9.3%, and for biocomposites from the 0/20 fraction, only 5.6%.

The greatest increase in thermal conductivity is observed when the compression of the specimens is increased. Although, after pressing 20%, the thermal conductivity of biocomposites is very low and close to the thermal conductivity of the wood waste itself, and in some cases even lower, but by further increasing the pressing every 20%, a sudden increase in thermal conductivity is observed. When comparing the thermal conductivity of the 80% pressed specimens with the 20% pressed specimens, an increase of even 43.3% is observed when using a 0/5 mm fraction filler, and a significantly lower thermal conductivity increase when using the 0/10 and 0/20 mm fillers, then there is an increase of 26.7% and 26.4%, respectively.

**Figure 8:**
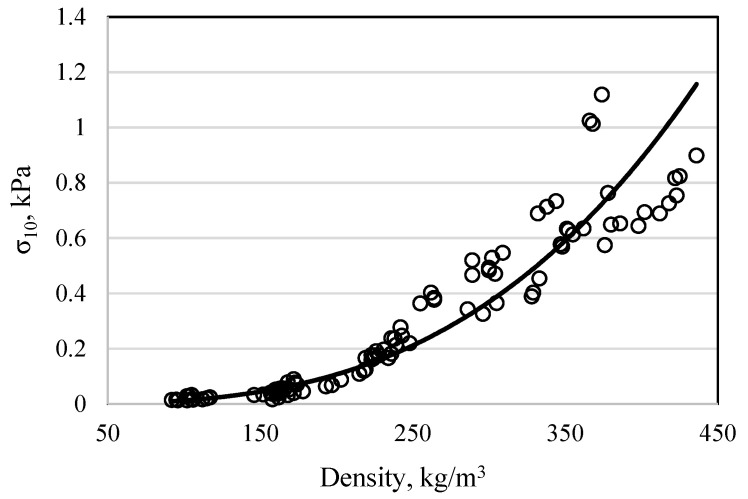
Dependence of the compressive stress of the biocomposite at 10% deformation on its density.

The dependence of compressive stress on the density of the biocomposites is presented in Figure 8. Analysis of the results shows that a 4 times increase in density resulted in an approximately 80 times increase in compressive stress. The obtained compression results were evaluated according to the size of the filler fraction, the compression level of the molding mixture, and the amount of sapropel in the mixture. The analysis of the results showed that using the same compression and the same filler fraction, and increasing the amount of sapropel three times, the compressive stress of the biocomposite increases by approximately 2 times, but the density increases only 1.06–1.30 times. The level of compression also has a significant influence on the compressive stress. Increasing the compression level by 20% increases the compressive stress by an average of 3.5 times. It can be assumed that there is compaction of filler particles through the largest air gaps. This assumption is confirmed when a different sized filler is used. Analysis of the results shows that when a fine fraction of 0/5 mm is used, the compressive strength is obtained up to 20 times higher than when using particles of a fraction of 0/20 mm and is approximately 15 times higher than when using particles of a fraction of 0/10 mm. The level of compression also has a significant effect on the release of the excess water from the mixture. Over 80% compression leads to intensive water release from the mixture; it is likely that some of the solid sapropel particles are also removed with the water.

Comparing the results of thermal conductivity (Figure 7) and compressive stress (Figure 8), it can be seen that when the density of the biocomposite reaches approximately 300 kg/m^3^, not only the compressive stress but also the coefficient of the thermal conductivity starts to increase suddenly. To obtain a biocomposite that has not only low thermal conductivity but also sufficient strength, the mentioned density limit is very important. Among the biocomposites at the density limit of 300 kg/m^3^, the biocomposites made from mixture compositions of 2-10–2-12 and 3-10–3-12 had the highest strength and the lowest thermal conductivity. The thermal conductivity of these biocomposites varied within very small limits of 0.0612 to 0.0659 W/(mK), while the compressive stress values varied approximately 2.5 times from 0.414 to 1.051 MPa. 

Different fillers and their preparation methods lead to different properties of biocomposites. For this reason, it is difficult to accurately compare the results obtained by different authors. The density of the biocomposites obtained with the sapropel binder also varies widely, from 157 to 540 kg/m^3^ [16,19,26]. In the work [19], the thermal conductivity of the biocomposites with wood particles was 0.06–0.061 and the compressive strength was 0.19–0.77 MPa, which is very close to the biocomposites with the same density as in our work.

In recent decades, biocomposites have been developed using various organic binders. A commonly used binder is a starch-based binder. Pundiene et al. [34] found that the compressive stress of the biocomposites with a starch-based binder varies from 0.49 to 1.4 MPa. The results obtained in [34] are similar to the results obtained in our work.

### 3.4. Structure of Biocomposite

**Figure 9:**
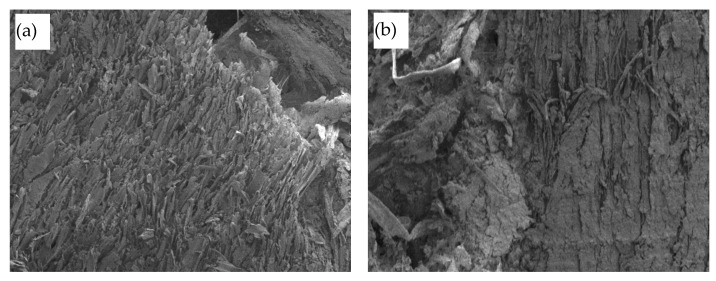

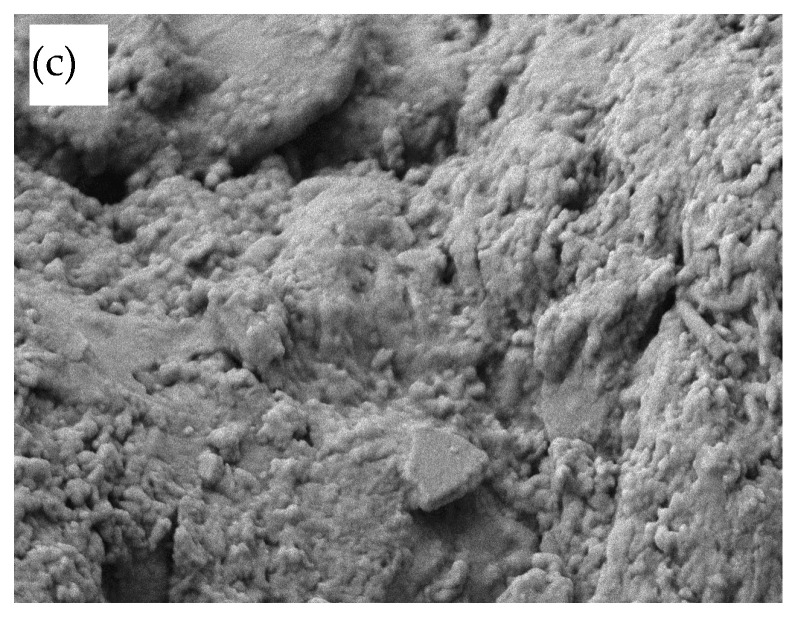
Structure of the biocomposite: (**a**) hardened sapropel on the rough surface of wood waste particle, magnification ×300; (**b**) hardened sapropel on the flat surface of wood waste particle, magnification ×300; (**c**) distribution of hardened sapropel on the surface of wood waste particles magnification ×5000.

The image of the structure of the prepared biocomposite is presented in Figure 9. The analysis of the structure allowed us to determine a different view in different parts of the wood waste particles. Figure 9a shows how the solidified sapropel is distributed over the uneven surface of the particle. The uneven surface of the particle was formed during the wood chipping process at the point of breakage of the wood particle. Since many small fibers are formed at the fracture site during the preparation of the mixture, a lot of sapropel accumulates between these fibers, which attaches to them during hardening. Figure 9b shows how hardened sapropel coats the smooth surface of a wood particle. During curing, the sapropel attaches to the surface of the wood particles and between the longitudinal grooves of the wood particles.

To clarify the principle of action of the sapropel during the formation of the biocomposite, a higher magnification was used (see Figure 9c). Higher magnification showed the formation of many discrete and very small zones of solidified sapropels on the wood particles in all cases. Since the water content in the used sapropel is more than 90%, very large contractions occur in the sapropel during curing, so the mass of the not yet hardened sapropel splits due to high stresses and is almost evenly distributed over the entire area of wood waste. In Figure 9c, sapropel zones bonded to wood particles are observed, which form a large number of contact zones. From the figure, it can be concluded that the contact areas are sufficiently strong, as no cracks or splits are observed.

## 4. Conclusions

Mechanically activating the sapropel increases its spread by more than 60%, obtains a homogeneous mass, and increases the tensile strength of the hardened sapropel more than twice compared to the mechanically unactivated sapropel.

Wood waste is crushed in a mill with sieves of different mesh sizes, and the resulting particles not only have different densities and thermal conductivity, but also change the ratio of particles of different sizes.

The thermal conductivity and compressive stress of the obtained biocomposites can be adjusted by changing the filler fraction, the amount of sapropel, or the level of compression. The level of compression of the biocomposite during its production has the greatest influence on the thermal conductivity and compressive stress of the biocomposite.

Laboratory biocomposite studies show good results, but in order to use sapropel for the industrial production of biocomposites, it is necessary to carry out studies related to moisture properties, dimensional stability, and durability, as well as to examine in detail the other possible sapropel processing options, such as curing with the help of microwaves, the partial drying of sapropel before use, the use of high pressure to remove water from sapropel, etc.

## Figures and Tables

**Table 1:** Characteristics of the sapropel used in the investigation.

Place of Sampling Sapropel	Water Content, Mass %	Content of Organic Matter, Mass %
Lake Kerėplis, the district of Trakai, Lithuania	93.1	95.8

**Table 2:** Description of the wood waste particles after milling.

Mesh Size of Mill Sieves, mm	Residue of Wood Waste Particles on Sieves, %						
	Sieve Mesh Size, mm						
	10	5	2.5	1.25	0.63	0.315	0
20	1.5	38.8	50.8	3.0	4.3	1.3	0.3
10	0	14.7	67.6	7.0	7.7	2.0	1.0
5	0	0	38.0	14.7	31.7	11.3	4.3

**Table 3:** The composition of the forming mixtures and their compression levels.

Mixture No.	Fraction of Used Wood Waste, mm	Content of Sapropel, %, from the Mass of Wood Waste	Compression Level, % of the Height of the Initial Mass of the Mixture
1-1	0–5	4	20
1-2		8	
1-3		12	
1-4		4	40
1-5		8	
1-6		12	
1-7		4	60
1-8		8	
1-9		12	
1-10		4	80
1-11		8	
1-12		12	
2-1	0–10	4	20
2-2		8	
2-3		12	
2-4		4	40
2-5		8	
2-6		12	
2-7		4	60
2-8		8	
2-9		12	
2-10		4	80
2-11		8	
2-12		12	
3-1	0–20	4	20
3-2		8	
3-3		12	
3-4		4	40
3-5		8	
3-6		12	
3-7		4	60
3-8		8	
3-9		12	
3-10		4	80
3-11		8	
3-12		12	

**Table 4:** Description of the consistency of the sapropel.

Number of Test Specimen	Activation Time, min	Consistency, mm
1	0	112
2	1	152
3	2	167
4	3	168

## Data Availability

The study did not report any data.
